# Understanding epistemic injustice among individuals with psychotic experiences: a scoping review protocol

**DOI:** 10.1136/bmjopen-2026-125288

**Published:** 2026-10-26

**Authors:** Raffaele Lavalle, Jodie Louise Russell, Lisa Bortolotti, Michael Larkin, Antonio Preti, Matthew Broome

**Affiliations:** 1 Department of Neuroscience ‘Rita Levi Montalcini’, University of Turin, Turin, Italy; 2 Institute for Mental Health, School of Psychology, University of Birmingham, Birmingham, UK; 3 Philosophy Department, University of Birmingham, Birmingham, UK; 4 Department of Neuroscience and Rehabilitation, University of Ferrara, Ferrara, Emilia-Romagna, Italy; 5 Institute of Health and Neurodevelopment, Aston University, Birmingham, West Midlands, UK; 6 Birmingham and Solihull Mental Health NHS Foundation Trust, Birmingham, UK

**Keywords:** MENTAL HEALTH, MEDICAL ETHICS, Schizophrenia & psychotic disorders

## Abstract

**Abstract:**

**Introduction:**

Epistemic injustice—namely testimonial and hermeneutical injustice—has increasingly been recognised as a critical concern within mental health research and practice, including in relation to psychosis. Despite the growing theoretical interest in this area, empirical evidence remains sparse, conceptually fragmented and methodologically heterogeneous. To date, no scoping or systematic review has examined how epistemic injustice is experienced by individuals with psychosis or psychotic-like experiences, or how it is addressed in the existing literature, thereby signalling a clear and currently unmet need for evidence synthesis.

**Methods and analysis:**

The present protocol outlines the methodology for a scoping review designed to systematically map the breadth, depth and characteristics of the available evidence concerning epistemic injustice in people with psychotic experiences. The review aims to: identify how epistemic injustice is conceptualised and described in relation to psychosis or psychotic-like experiences; summarise the findings regarding the experience of epistemic injustice in people with psychotic experiences; explore how the literature addresses complex issues such as impaired insight, alterations in intersubjective experience of reality, involuntary treatment and social and political marginalisations; examine the implications for clinical practice, training, service organisation and policy while identifying gaps warranting further empirical investigation. The protocol was developed following the Joanna Briggs Institute (JBI) methodology for scoping review protocols and reported in accordance with the Preferred Reporting Items for Systematic Reviews and Meta-Analyses (PRISMA)-Protocols (PRISMA-P) checklist. The review will be conducted in accordance with JBI methodology for scoping reviews and reported in line with the PRISMA extension for Scoping Reviews (PRISMA-ScR). A comprehensive search strategy will be implemented across major electronic databases, complemented by reference list screening. Two reviewers will independently screen, select and extract data, with disagreements resolved through discussion and when required, consultation with additional reviewers. Data will be charted using an iterative approach and synthesised through descriptive, narrative or thematic techniques. Eligible sources will include quantitative and qualitative empirical studies, case reports and case series and contributions including first-person accounts of epistemic injustice among individuals with psychotic experiences. Following protocol registration, the search strategy was finalised on 26 June 2026 and title/abstract screening has since commenced. No results are reported at this stage; these will be presented in full in the completed scoping review.

**Ethics and dissemination:**

Findings will provide the first comprehensive mapping of this emerging field and inform future empirical research, mental-health education and policy development grounded in epistemic justice. Results will be disseminated through peer-reviewed publication, presentations at departmental seminars and scientific conferences, and a policy brief for policymakers, healthcare providers and other stakeholders, outlining actionable recommendations to address the challenges identified. This protocol was registered on the Open Science Framework on 18 June 2026 (OSF; DOI: https://doi.org/10.17605/OSF.IO/XSD58).

**STRENGTHS AND LIMITATIONS OF THIS STUDY**

- This protocol was developed in accordance with the Joanna Briggs Institute methodology for scoping reviews and reported according to the Preferred Reporting Items for Systematic Reviews and Meta-Analyses (PRISMA)-Protocols (PRISMA-P) checklist. The subsequent review will be reported in accordance with the PRISMA extension for Scoping Reviews (PRISMA-ScR) guidance, promoting methodological transparency and reproducibility.
- The protocol outlines a comprehensive and multidisciplinary search strategy, including multiple biomedical, psychological, philosophical and nursing databases, grey literature sources, backward citation searching and no language restrictions, with the aim of maximising the identification of relevant evidence.
- The protocol prespecifies rigorous procedures for study selection, data charting and conflict resolution, including independent screening and data extraction by two reviewers with consensus procedures involving additional reviewers when required, thereby enhancing methodological reliability.
- The protocol adopts a broad and interdisciplinary approach to evidence selection, encompassing empirical, theoretical and first-person contributions from mental health, philosophy, psychology and related disciplines, thereby enabling a comprehensive mapping of this emerging field.
- The absence of planned critical appraisal and quantitative synthesis (eg, meta-analysis), consistent with the scoping review methodology, will limit the ability of the completed review to draw conclusions about the quality of the evidence or the magnitude of observed effects.

## Introduction

 The philosopher Miranda Fricker first proposed the concept of epistemic injustice,^1^ to designate a specific form of injustice related to how credibility, belief, meanings are allocated.^2^ Epistemic injustice—intertwined, though distinct, with other *tòpoi* of psychiatric literature including stigma, negative stereotyping or sanism^3^—broadly refers to a kind of harm someone suffers in their capacity as a knower. This harm is perpetuated through *identity prejudices*, namely, particular biases against the social categories one belongs to, for example, racialised minorities or marginalised genders. In other words, identity prejudices structurally and unjustly influence *who* is perceived to know *what* such that it disadvantages, and disproportionately harms, particular groups of people.

Fricker elaborates on this concept by identifying two main types of epistemic injustice: testimonial and hermeneutical injustice. Testimonial injustice arises ‘when prejudice causes a hearer to give a deflated level of credibility to a speaker’s word’ (p. 1)^1^—the so-called *credibility-deficit*. Such deficits cause harm most evidently when critical information to someone’s health, for example, is not given sufficient credibility due to the identity of the knower. Later contributions have further developed and critically refined this framework.^3^ For example, Dotson^4^ identified two other types of testimonial injustice: ‘testimonial smothering’, a form of self-censorship prompted by anticipated dismissiveness and ‘testimonial quieting’, which occurs when prejudicial credibility deficits lead to a systematic devaluation of a person’s contributions.^2^ Hermeneutical injustice, on the other hand, refers to an unfair or unequal access or distribution of epistemic resources. The harm here becomes manifest ‘at a prior stage, when a gap in collective interpretive resources puts someone at an unfair disadvantage when it comes to making sense of their social experiences’ (p. 1).^1^

Mental-health settings have increasingly been recognised as contexts in which epistemic injustice may be particularly salient,^5^ possibly even more than in ‘physical’ health contexts.^6^ As Lisa Bortolotti and Broome clearly explain in their seminal book on epistemic injustice in mental healthcare, individuals accessing psychiatric services are often ‘already in a vulnerable position’ (p. VI), facing a ‘threat to’ their ‘sense of agency’ (p. VI), inherent in seeking help from technical experts.^7^ Within the broader healthcare system, structurally embedded power asymmetries and negative stereotypes about service users have likewise been identified as contributing to forms of epistemic marginalisation.^8 9^ How mental-health professionals position themselves in clinical encounters can therefore raise significant concerns regarding further disempowerment and epistemic subordination.^7^ Indeed, mutual trust is considered a central aspect of a therapeutic relationship.^10^ From this perspective ‘erosion of trust’ and ‘dysfunctional patterns of trust/distrust’ (p. 57) have been theorised as key mechanisms underlying epistemic injustice.^11^

Beyond the general concerns about epistemic injustice in psychiatric practice, recent literature has also examined how it manifests in groups of individuals with specific psychopathological and neurodevelopmental conditions, including obsessive–compulsive disorder,^12^ depression,^13^ borderline personality disorder,^14^ autism^15^ and eating disorders.^16^

A growing body of research has also begun to investigate epistemic injustice in the context of psychosis. Belvedere-Murri *et al*^17^ highlight how individuals with psychosis may be regarded as globally non-credible, even when expressing views unrelated to delusional or hallucinatory content. In other words, they may be assumed to ‘lack epistemic agency altogether’ (p. VI).^7^ Sanati and Kyratsous^18^ also discuss how ‘attributions of irrationality, bizarreness, and incomprehensibility’ (p. 479), as well as stereotypes—for instance portraying individuals as manipulative—significantly contribute to testimonial injustice in people with delusions. At the same time, various forms of hermeneutical injustice are likely to affect the lived experience of people with psychosis, for instance due to the ‘complex and often ineffable nature of psychotic experiences’ (p. 52),^17^ particularly in clinical voice-hearers.^19^ Patients with psychosis, especially inpatients, may additionally be victims of hermeneutic injustice due to automatic exclusion from some research studies because of the perceived vulnerability of this population by ethical review boards.^20^

With the typical peak onset of schizophrenia-spectrum disorders occurring in the late 20s for women and the early to mid-20s for men,^21^ the substantial prevalence of this condition in young people may further compound experiences of epistemic injustice, especially during the transition between child/adolescent and adult mental health services—a period increasingly recognised as neurodevelopmentally and organisationally sensitive.^22 23^ Indeed, widespread stereotypes regarding young people have been characterised, including their being ‘lazy and immature, to lack resilience, and to be attention-seeking’ (p. 24),^24^ which could prevent them from an empathic and curious listening by others.^25^ It is easy to think, given these premises, how epistemic injustice may represent a concrete risk for young people,^24 26^ especially in the case of severe mental illness^27^ or unusual beliefs and experiences^28^ such as hearing voices.^19^ The processes of trying to be taken seriously and have one’s voice heard are complicated further by the fact that according to some epidemiological data this population appears to be disproportionately represented by individuals from Black, Asian and Minority Ethnic backgrounds (eg, ^29^). Many young people with psychosis, then, may experience additional prejudice against their testimony on the basis of their race or ethnicity, for example, as well as their youth. Young people with psychosis are therefore likely to experience epistemic injustice as a result of multiple features of their identities.

In this regard, the concept of intersectionality, developed from Black feminist thought,^30–33^ captures these phenomena. While Collins^34^ acknowledges that intersectionality has no strict definition, it is generally agreed that, at its foundation, intersectionality captures the idea that we are not treated by institutions or other people on the basis of *singular* aspects of our identities, but, instead, these aspects compound; we are often treated by others on the basis of multiple aspects of our identity coinciding. Taking stock of these considerations, the intersection between identity, the lived experience of psychosis and epistemic injustice appears to raise issues of particular complexity, and perhaps even uniqueness of such cases.

At the same time, clinical experience with individuals with psychosis suggests that interpreting epistemic asymmetries solely in terms of injustice may be overly simplistic. According to the International Classification of Diseases 11th Revision, schizophrenia or other psychotic disorders are characterised by impaired reality testing,^35^ a feature that may immediately undermine the credibility attributed to individuals with psychosis. Additionally, poor insight—traditionally viewed as a core feature of psychosis^36 37^—may introduce an additional layer of epistemic vulnerability, particularly if invoked to question individuals’ reliability or self-understanding. Lastly, clinical severity may also expose service users to involuntary interventions, ranging from compulsory admissions to subtler forms of informal coercion,^17^ further affecting their epistemic standing. These considerations raise an important question: should such dynamics and conceptualisations be regarded purely as further forms of epistemic injustice, or do some forms of epistemic vulnerability instead reflect aspects of psychopathological suffering that cannot be fully captured by the notion of undue harm?

These issues may already indicate possible directions. As stated above, it is important to avoid the risk of implicitly endorsing a view that would amount to a complete deprivation of a person’s epistemic agency.^7^ Even mechanistic accounts, such as the aberrant salience model,^38–40^ tend to frame psychosis in terms of altered experience of meaning. However, psychotic experiences—including alterations, according to intersubjective standards, in how individuals experience reality or a reduced insight into illness—are not all-or-nothing phenomena and do not affect the entirety of a person’s experiential field. According to this non-totalising nature of psychotic experience, as already established in some national legislations (eg, Law 180 in Italy), the implementation of involuntary treatments should be strictly conditioned on the presence of severe psychic alterations requiring urgent therapeutic intervention—although refused by the person concerned—that cannot be addressed in a timely and adequate manner in extrahospital settings, with the patient’s health as the central concern. At the same time, keeping the person’s well-being at the centre requires resisting the reduction of the complexities of care to one particular lens of analysis, while remaining open to concerns about epistemic injustice.

In light of these challenges, developing a more nuanced understanding of subjective experience becomes essential for engaging with suffering individuals in a manner that is both person-centred and epistemically just. Phenomenological insights,^3 17^ particularly those from critical phenomenology,^41^ have therefore been proposed as valuable resources for framing subjective experience within a structural and person-centred approach.

Despite the growing theoretical interest in this area, empirical research remains scarce^42^ and largely non-systematised. Much of the literature consists of conceptual analyses, with limited systematic examination of how epistemic injustice is experienced by people with psychosis in real-world contexts.

To the best of our knowledge, and at the time of registration of the present protocol, a preliminary examination of the main databases included in this review did not identify any scoping or systematic reviews specifically addressing the experience of epistemic injustice in the context of psychosis or psychotic-like experiences, highlighting a clear gap in the existing literature. This paucity and heterogeneity warrant further investigation, given the relevance of the topic. Indeed, examining epistemic injustice in the experience of people with psychosis is a matter of pressing contemporary relevance, with significant implications at multiple levels: ethical, for mental health professionals; pragmatic, for the organisation of mental health services; and political, for legislative frameworks. It is therefore essential that legal and institutional structures support efforts by practitioners and mental-health services to orient care toward epistemically just approaches that respect human rights and aim to provide, with professional integrity, the best possible care.

## Objectives

From a research perspective, our goal is to explore and map the existing evidence, as well as its depth, breadth and nature, in the emerging field concerning the experience of epistemic injustice in individuals with psychosis or psychotic-like experiences. We also aim to stimulate further empirical work in this area by identifying current knowledge gaps, which this review seeks to map. Moreover, we intend to clarify whether, and in what ways, the existing literature addresses the most complex and problematic issues related to epistemic injustice in people with psychotic experiences—for instance, impaired insight, disruptions in intersubjective experience of reality, involuntary treatment, but also social and political marginalisations. By informing empirical research in this field, we hope to contribute to the development of a sufficiently robust body of evidence that may eventually support future systematic reviews and meta-analyses. At the same time, while remaining consistent with the scoping review design and limitations, we adopt a pragmatic orientation and aim to provide mental health decision-makers, as well as those involved in mental health education, with valuable insights to inform policies and care practices grounded in the principles of epistemic justice, as advocated by many of the authors we cite.^6 19^

It is on the basis of these methodological premises that we opted for a scoping review design. Indeed, a scoping review is particularly appropriate given the exploratory nature of the topic and the heterogeneity in terms of content and methodology of the available evidence, as it allows for the mapping of key concepts, types of evidence and knowledge gaps without restricting inclusion to narrowly defined study designs.^43^ This design also emerged—based on the information we provided—as one of the options suggested by an existing online tool for selecting the most appropriate review type.^44^ Therefore, this protocol refers to a scoping review that will be entitled ‘Understanding epistemic injustice among individuals with psychotic experiences: a scoping review’.

## Review questions

How is epistemic injustice experienced by individuals with psychosis or psychotic-like experiences, and how are its manifestations described in the literature?

The following subquestions are included:

How does the existing literature address complex issues related to epistemic injustice in psychosis, such as social and political marginalisations, impaired insight, disruptions in intersubjective experience of reality and involuntary treatment?

What implications of epistemic injustice in psychosis are reported for clinical practice, mental health education, service organisation, policy and on an individual-level?

What gaps in the empirical evidence are identified?

Our eligibility criteria and overall methodological approach will be guided by the Population–Concept–Context framework, as recommended in the Joanna Briggs Institute (JBI) and Preferred Reporting Items for Systematic Reviews and Meta-Analyses extension for Scoping Reviews (PRISMA-ScR) guidelines.^43 45–47^

## Eligibility criteria

### Population

Our review will include contributions examining epistemic injustice in populations with psychosis or psychotic-like experiences. We will not specify any inclusion criteria concerning the characteristics of participants with psychotic experiences in the included works—for example, gender, age, ethnicity, mental health status, specific psychopathological condition, diagnostic or assessment tools, or any other relevant factor. In line with a framework informed by epistemic justice, we have decided not to exclude any particular segment of the general population.

### Concept

The concept of interest in this scoping review is epistemic injustice as experienced by individuals with psychosis or psychotic-like experiences. Epistemic injustice, as originally defined by Fricker,^1^ refers to wrongs done to individuals in their capacity as knowers and includes testimonial injustice, whereby a speaker’s credibility is unjustly deflated due to prejudice and hermeneutical injustice, which arises when structural gaps in interpretive resources render certain experiences difficult to understand or articulate. In the context of psychosis, epistemic injustice may manifest in multiple and inter-related ways. These include the unjustified discounting of individuals’ epistemic credibility, particularly in relation to delusional or hallucinatory experiences but also extending to areas unrelated to psychopathological content. It may further involve difficulties in mutual understanding and epistemic vulnerabilities associated with psychopathological forms of vulnerability, including disruptions in shared interpretive frameworks and challenges in articulating and recognising lived experience. Additional dimensions relevant to this review include experiences of reduced epistemic agency, the role of insight in shaping credibility attributions and the impact of clinical power asymmetries, including coercive or involuntary treatment practices. These processes may also be shaped by intersecting identity factors, such as race and age, which may compound epistemic disadvantage in line with an intersectional framework.^30–34^

### Context

No restrictions will be applied concerning the context in which participants are situated: studies involving individuals engaged with health services, as well as those conducted with participants not receiving clinical care, will both be included. Furthermore, as stated in the Participants section, no limitations will be imposed regarding geographical location or any specific gender, cultural, ethnic or social factors. Finally, the database searches will be conducted with a temporal scope starting from 2007, the year in which Fricker first conceptualised epistemic injustice.^1^ In accordance with the values underpinning epistemic justice, we have decided not to impose restrictions on the contexts in which such experiences occur.

### Types of evidence sources

Studies investigating epistemic injustice in populations with psychosis or psychotic-like experiences will be included, encompassing quantitative and qualitative empirical studies, case reports and case series, and contributions including first-person accounts of epistemic injustice. Undergraduate and master’s dissertations, conference abstracts (poster or oral presentations), study protocols and other works not meeting the predefined eligibility criteria in terms of content and format will be excluded to ensure adequate methodological robustness. However, doctoral theses will be included. In cases of duplicate findings arising from the inclusion of both primary studies and evidence syntheses, primary studies will be excluded when they are encompassed within an included evidence synthesis, unless they provide additional data not reported in the synthesis.

All eligibility criteria detailed above, as well as their interpretation, were agreed on by all authors.

## Methods

The present scoping review will be conducted in accordance with the JBI methodology for scoping reviews^43 45^ and reported according to the PRISMA-ScR.^47^ The present protocol has also been prepared following these guidelines^46^ and reported according to the PRISMA-Protocols Checklist^48^ (see online supplemental appendix III), since, to the best of our knowledge, no specific checklist for scoping review protocols is currently available. Furthermore, other scoping review protocols, identified as aligned with JBI methodologies,^46^ have been consulted.^49–51^ This protocol was prospectively registered on the Open Science Framework on 18 June 2026 (OSF; DOI: https://doi.org/10.17605/OSF.IO/XSD58).

### Reviewers positionality

Given the interpretive and interdisciplinary nature of the subject matter, we report the disciplinary backgrounds of the review team for transparency. The two primary reviewers (RL, JR), responsible for screening, charting and pilot calibration, are a resident physician in psychiatry and a philosopher (PhD) with an academic research background, respectively. The four senior authors (MB, AP, LB, ML) are university professors: two psychiatrists whose research interests span biologically oriented and humanities-oriented approaches to mental illness, with a particular focus on phenomenology; one philosopher; and one psychologist. This composition reflects a deliberate effort to balance clinical and philosophical/humanities perspectives throughout the review process, given that judgements about what constitutes epistemic injustice in the context of psychosis are not theory-neutral and may be inflected by disciplinary training.

### Search strategy

Searches will be conducted in MEDLINE, Web of Science, PsycINFO, CINAHL and PhilPapers, covering all records available from 2007 until June 2026. Although these databases index some unpublished materials, the search will also be extended to CORE to further enhance the coverage of grey and unpublished literature. Search strings developed by authors, in consultation with an experienced university research librarian, will include combinations of terms and keywords related to ‘epistemic injustice’, ‘psychosis’ and associated concepts. Details on the search strings can be found in online supplemental appendix I.

No language restriction will be applied at the search-string level, in order to minimise language bias in study identification. Titles and abstracts retrieved in languages read fluently by the research team (English, Italian, French and Spanish) will be screened directly by two independent reviewers according to the standard eligibility criteria. For records in languages not read by any team member, a two-step procedure will be applied. First, titles and abstracts will be translated using a machine-translation tool to allow a preliminary judgement of eligibility. Records judged as potentially eligible, or where eligibility cannot be confidently excluded from the translated abstract, will proceed to full-text retrieval and machine-translated full-text screening; where the translated full text still leaves eligibility unresolved or where inclusion is confirmed and data extraction is required, translation support will be sought from a native or fluent speaker (institutional colleague or professional translation service, as available) before a final inclusion/exclusion decision is made.

Additional relevant records will be identified through reference list checking (backward citation searching), by examining the reference lists of the included works. Forward citation searching of the included papers will also be implemented. Furthermore, any additional contributions known to the authors and relevant to the inclusion criteria—but not retrieved by the search strategy—will be considered for inclusion. Citation management will be conducted using Mendeley reference manager software (https://www.mendeley.com/).

A preliminary literature search was conducted, and last updated, on 26 June 2026, after the protocol registration and title and abstract screening commenced on the same date. Full-text eligibility assessment is anticipated to begin in October 2026. Data extraction/charting and data analysis/narrative synthesis are anticipated to follow in rapid succession, with completion and submission for publication anticipated by early 2027. As detailed below, any substantive deviation from this anticipated timeline will be documented and reported transparently.

### Study/source of evidence selections

All retrieved records will be independently screened by two reviewers (RL and JR) based on title and abstract to assess their relevance to the review objectives and eligibility criteria. Any discrepancies will be resolved through discussion, with consultation of four more experienced authors (MB, AP, LB and ML) when necessary, until consensus is reached. Full texts of all potentially eligible studies will then be independently assessed against the predefined inclusion criteria by the same two reviewers (RL and JR). Any disagreements will again be resolved through discussion, with input from the four more experienced authors (MB, AP, LB and ML) when required, until consensus is achieved. Prior to formal screening and full-text selection, a pilot screening of a subset of records (eg, 5%) will be conducted by the two primary reviewers (RL and JR) to ensure consistency in the application of eligibility criteria. Inter-rater agreement at the pilot stage will be quantified using Cohen’s kappa. A threshold of κ≥0.70 will be considered adequate to proceed to full screening; if agreement falls below this threshold, eligibility criteria will be clarified and discussed between RL and JR, with input from the four more experienced authors (MB, AP, LB and ML) where necessary and the pilot will be repeated on a new subset of records before full screening resumes. Duplicate removal, title/abstract screening and full-text assessment will be conducted using Rayyan (https://www.rayyan.ai/). Manual checking will be performed after the automated duplicate-removal process to ensure accuracy. In keeping with the iterative nature of scoping reviews, the search strategy and eligibility criteria may be refined and iteratively expanded during the review process should new relevant concepts, terms or sources of evidence emerge during screening or study selection.

### Data extraction and charting

Two reviewers (RL and JR) will independently extract and chart relevant data into Microsoft Excel spreadsheets, in accordance with the stated aims and research questions of the scoping review. We will extract the following variables: first author(s); year of publication; location; aims/purpose; journal; study design and methodology; disciplinary field (eg, philosophy, psychiatry, psychology). For empirical sources involving human participants, sociodemographic and clinical characteristics of the sample will additionally be extracted. This field will be marked as not applicable for conceptual/theoretical works, for which the theoretical framework and central thesis or argument will be extracted instead; for first-person/experiential accounts, sociodemographic information will be recorded only where self-disclosed by the author within the source, together with the author’s self-identified experiential standpoint where stated (eg, person with lived experience of psychosis, clinician, family member). A draft charting table can be found in the online supplemental appendix II of the present protocol. However, this table may be further revised during the review process, in line with the iterative nature of scoping reviews. The data charting form will be piloted on a subset of included studies (eg, 5–10%) to ensure clarity and consistency among reviewers prior to full data extraction. As for the selection stage, inter-rater agreement at this pilot stage will be assessed using Cohen’s kappa, with a threshold of κ≥0.70 required before proceeding to full data extraction; where this threshold is not met, the charting form will be revised and the pilot repeated. The data charting rules and coding/recording instructions for each source will be documented in a shared Excel file accessible to all reviewers. Any missing or unclear data will be recorded as missing in the extraction form, and the authors of the included studies will not be contacted to obtain further information. Any discrepancies will be resolved through discussion, with input from four additional reviewers (MB, AP, LB and ML) when required, until consensus is reached. In accordance with the latest JBI guidance for scoping reviews,^43 45^ no critical appraisal or risk of bias assessment will be conducted.

### Data analysis and presentation

Prior to analysis, all charted data will be cross-checked and cleaned to ensure accuracy, consistency and completeness. This will involve identifying missing values, harmonising terminology and removing any remaining duplicates or formatting inconsistencies. Data cleaning and management will be conducted using Microsoft Excel, which will also be used for data organisation and preliminary analysis.

The results section of our scoping review will include a first subsection mainly dealing with the search strategy and selection process, and a second subsection presenting the main information or findings relevant to the objectives and research questions of the scoping review.

A PRISMA 2020 flow diagram will be used to document the study selection process, reporting the number of records identified, screened, excluded and included, together with the reasons for exclusion at the full-text stage. Furthermore, the study selection process will also be reported narratively, in accordance with the PRISMA-ScR guidelines.^47^ Information on all studies excluded at the full-text assessment stage will be reported in an appendix, together with the specific reasons for their exclusion.

Given the scoping nature of the review, data will be presented using a descriptive and narrative approach, with findings mapped against the review objectives. Quantitative data will be summarised using descriptive statistics (eg, simple percentages or frequency distributions) where appropriate, while qualitative and theoretical findings will be organised narratively and/or thematically.

Results will also be reported through a combination of visual representations (charts, figures, tables, maps or diagrams) to support interpretation of the evidence. Where possible, the interpretation of findings may be informed by consultation with relevant stakeholders or experts by experience, in order to enhance interpretative depth.

Each data item will be handled as follows: bibliographic and methodological information will be summarised in descriptive tables; sample characteristics will be tabulated to provide an overview of the populations studied; and main findings will be presented narratively and/or thematically in relation to epistemic injustice and psychosis.

### Protocol amendments

Any deviations from the procedures described in this protocol will be documented and made transparent. Substantive amendments (eg, changes to eligibility criteria, search strategy or charting variables) will be logged with the date of the change, a rationale and the stage of the review at which it occurred, and will be reported in the final manuscript. The OSF registration will be updated accordingly

## Patient and public involvement and engagement

Members of patient groups or the public were not involved in the development of this protocol. However, researchers plan to consult the University of Birmingham’s Youth Advisory Group on the findings of the review, on ways to raise awareness of epistemic injustice and to coproduce plain language summaries of the results that are accessible to individuals with varying levels of literacy and education. These summaries will distil key findings into clear and understandable language, making them accessible to service users, caregivers, policymakers and the general public. These will be disseminated through the Epistemic Injustice in Healthcare blog: a publicly available platform summarising research in the area of epistemic injustice.

We also plan to develop the policy brief, alongside other amelioration strategies, with the Youth Advisory Group, with the intention of implementing strategies to mitigate epistemic injustice among people with psychosis.

## Discussion

This scoping review protocol outlines a structured and methodologically rigorous approach to examining how epistemic injustice is experienced by individuals with psychosis or psychotic-like experiences across diverse contexts and forms of evidence. By adopting the JBI–PRISMA methodology and an intentionally inclusive strategy, the review aims to map the breadth and nature of existing knowledge while identifying conceptual, methodological and empirical gaps in this emerging field. Given the ethical and clinical relevance of epistemic justice in mental healthcare—and the particular vulnerabilities faced by people with psychotic experiences—this review has the potential to inform future research, support more epistemically sensitive clinical practices and guide educational and policy initiatives. Ultimately, by clarifying how epistemic injustice manifests in the lives of those with psychosis, the review seeks to contribute to improving mental healthcare systems and fostering conditions in which service users’ voices and epistemic agency are appropriately considered and taken into account.

## Ethics and dissemination

As this is a scoping review, ethics approval is not required. As outlined above, all authors contributed to the development of the search strategy and collectively agreed on the eligibility criteria and their interpretation. RL and JR will lead the screening of titles/abstracts, full-text assessment, and data extraction and charting. MB, AP, LB, and ML will be consulted as needed to reach consensus at any stage of the review process and will provide substantial methodological and conceptual support.

The anticipated CRediT author contributions are as follows, although minor adjustments may occur as the review progresses: RL: Protocol Conceptualization; Review Conceptualization; Methodology; Formal Analysis; Investigation; Resources; Data Curation; Writing - Original Draft; Visualization. Guarantor. JR: Protocol Conceptualization; Review Conceptualization; Methodology; Formal Analysis; Investigation; Resources; Data Curation; Writing - Original Draft; Visualization. MB: Protocol Conceptualization; Review Conceptualization; Methodology; Writing - Review & Editing; Supervision; Project Administration; Funding Acquisition. LB: Protocol Conceptualization; Review Conceptualization; Methodology; Writing - Review & Editing; Supervision. ML: Protocol Conceptualization; Review Conceptualization; Methodology; Writing - Review & Editing; Supervision. AP: Protocol Conceptualization; Review Conceptualization; Methodology; Writing - Review & Editing; Supervision. Where feasible, the interpretation of findings may be guided by input from relevant stakeholders or experts by experience to enrich the depth of interpretation.

We plan to publish the scoping review in a peer-reviewed journal and to present its results during internal departmental seminars or at scientific conferences. A policy brief summarising the key findings and recommendations arising from the research will be developed and distributed to policymakers, healthcare providers, and relevant stakeholders. The brief will highlight actionable insights and strategies for addressing the challenges identified in the study, with the aim of influencing policy development and service provision.

## Supplementary material

10.1136/bmjopen-2026-125288 online supplemental appendix 1

10.1136/bmjopen-2026-125288 online supplemental appendix 2

10.1136/bmjopen-2026-125288 online supplemental appendix 3
