# Supplementary material for: Understanding epistemic injustice among individuals with psychotic experiences: a scoping review protocol
Source: BMJ Open. 2026 Oct 26;16(10):e125288. doi: 10.1136/bmjopen-2026-125288 (PMC13711525; doi:10.1136/bmjopen-2026-125288)
Supplement: Supplementary file 1 [file bmjopen-16-10-s002.pdf]

First author(s)	Year of publicatior	Location	Aims/purpose	Journal	Study design and methodolo	Disciplinary field	Main findings
