# Supplementary material for: Understanding epistemic injustice among individuals with psychotic experiences: a scoping review protocol
Source: BMJ Open. 2026 Oct 26;16(10):e125288. doi: 10.1136/bmjopen-2026-125288 (PMC13711525; doi:10.1136/bmjopen-2026-125288)
Supplement: Supplementary file 2 [file bmjopen-2026-125288.reviewer_comments.pdf]

PEER REVIEW HISTORY

BMJ Open publishes all reviews undertaken for accepted manuscripts. Reviewers are asked to complete a checklist review form and are provided with free text boxes to elaborate on their assessment. These free text comments are reproduced below.

ARTICLE DETAILS

Title (Provisional)

Understanding epistemic injustice among individuals with psychotic experiences: a scoping review protocol

Authors

Lavalle, Raffaele; Russell, Jodie Louise; Bortolotti, Lisa; Larkin, Michael; Preti, Antonio; Broome, Matthew

---

VERSION 1 - REVIEW

**Reviewer**	**1**
**Name**	**De Berardis, Domenico**
**Affiliation**	**University "G. D'Annunzio" Chieti**
**Date**	**09-Aug-2026**
**COI**	**None**

As a protocol the manuscript reports no findings, which is appropriate. The methods are sound: JBI guidance with PRISMA-ScR reporting, a PCC framework, prospective OSF registration, five databases plus CORE, coverage from 2007 with no language restriction, dual independent screening and charting with consensus involving four senior authors, Rayyan and Mendeley for record management, no critical appraisal, and descriptive, narrative and thematic synthesis. The rationale is convincing and the gap credible. Nothing compromises scientific validity, so I recommend Minor revision.

1. The introduction reads very well, and the two clinically most delicate passages could be anchored a little more firmly in the psychiatric literature. Where the authors discuss the early peak of onset and the consequences for young people, a reference to work on early onset psychosis and on the transition between child, adolescent and adult services would support the argument, for instance https://doi.org/10.3390/brainsci11020275. Where they discuss alterations in the intersubjective experience of reality, the literature on aberrant salience offers a mechanistic account of how meaning is attributed to neutral stimuli and would enrich the framing, for instance https://doi.org/10.3389/fpsyt.2023.1343884. Neither source appears in the current reference list. I would also ask that the sentence invoking ICD-

11 for impaired reality testing carry its own citation, since the neighbouring onset data rest on reference 20.

2. In the objectives the authors propose to offer preliminary insights into how common this experience is. Given the heterogeneity of eligible sources, the inclusion of theoretical and first person contributions and the deliberate absence of critical appraisal, frequency cannot be estimated. Rephrasing this aim as mapping the extent, range and nature of the available evidence would keep the objectives fully consistent with the chosen design.

3. The search deserves fuller reporting. The string in Appendix I follows Ovid syntax, so please state the interface used for each database and supply the adapted strings as supplementary material, with the planned or actual search dates. Very broad terms such as lived experience, patient voice, dismissal and objectification will lower precision, and silencing appears twice within the same string, so a brief justification and a cleanup would help. Adding Embase or Scopus, forward citation searching, and a procedure for records in languages the team does not read would strengthen coverage.

4. For selection and charting, it would help to prespecify the level of agreement expected at the pilot stage before full screening proceeds, and to clarify how the charting form will accommodate conceptual and first person works, for which the field on sociodemographic characteristics of the sample does not apply. Given the interpretive nature of the subject, a brief statement on reviewer disciplinary background and positionality would add welcome transparency.

5. Finally, a few reporting details. There is no protocol specific extension of PRISMA-ScR, so the mention of PRISMA-ScR P is better replaced by the JBI best practice guidance already cited as reference 39. As BMJ Open asks protocols to report study dates, please add the anticipated start and completion of screening, extraction and synthesis, together with the OSF registration date and a policy for documenting deviations. A completed PRISMA-ScR checklist would be welcome, and a language check is advisable, since slips such as expecially, disproportionaly and an experienced university research remain.

VERSION 1 - AUTHOR RESPONSE

We sincerely thank the reviewer for the thoughtful and constructive feedback provided on our manuscript. We have carefully evaluated each of the comments and addressed the issues raised by making the appropriate revisions to the manuscript. In the following, we respond to each comment individually.

- "The introduction reads very well, and the two clinically most delicate passages could be anchored a little more firmly in the psychiatric literature. Where the authors discuss the early peak of onset and the consequences for young people, a reference to work on early onset psychosis and on the transition between child, adolescent and adult services would support the argument, for

instance https://doi.org/10.3390/brainsci11020275. Where they discuss alterations in the intersubjective experience of reality, the literature on aberrant salience offers a mechanistic account of how meaning is attributed to neutral stimuli and would enrich the framing, for instance https://doi.org/10.3389/fpsyt.2023.1343884. Neither source appears in the current reference list. I would also ask that the sentence invoking ICD-11 for impaired reality testing carry its own citation, since the neighbouring onset data rest on reference 20."

We thank the reviewer for this thoughtful comment. In response, we have added a sentence highlighting the particular sensitivity of the transition from childhood and adolescence to early adulthood, supported by relevant references. We have also included a brief reference to the mechanistic model of aberrant salience, which may provide a biological framework for understanding the altered attribution of meaning observed in psychosis; this statement is supported by relevant references. Finally, we have added the previously missing citation of the ICD-11.

- "In the objectives the authors propose to offer preliminary insights into how common this experience is. Given the heterogeneity of eligible sources, the inclusion of theoretical and first person contributions and the deliberate absence of critical appraisal, frequency cannot be estimated. Rephrasing this aim as mapping the extent, range and nature of the available evidence would keep the objectives fully consistent with the chosen design."

We thank the reviewer for these suggestions. In response, we have removed the specific reference to the frequency of experiences of epistemic injustice among individuals with psychosis as one of the objectives of our scoping review.

- "The search deserves fuller reporting. The string in Appendix I follows Ovid syntax, so please state the interface used for each database and supply the adapted strings as supplementary material, with the planned or actual search dates. Very broad terms such as lived experience, patient voice, dismissal and objectification will lower precision, and silencing appears twice within the same string, so a brief justification and a cleanup would help. Adding Embase or Scopus, forward citation searching, and a procedure for records in languages the team does not read would strengthen coverage."

We appreciate this fruitful comment, which allowed us to strengthen several aspects of our search strategy and reporting. As suggested, we have updated Appendix I to include the full search string for each database employed, specifying the interface used and the date of our first preliminary searches, conducted after protocol registration on OSF; planned timing for further review steps is also reported in the full text. We have also corrected the repetition of the term "silencing" within the search string, which was a technical oversight, and revised the string accordingly. Following the reviewer's suggestion, we will implement forward citation searching as an additional step to strengthen the coverage of relevant records. In addition, we have introduced a dedicated procedure for handling

records in languages not read by the team, now detailed in the Methods section, "Search strategy" paragraph. Concerning the broad terms flagged by the reviewer (lived experience, patient voice, dismissal, objectification) and the possible inclusion of additional databases such as Embase or Scopus, we would respectfully note that our current strategy reflects a deliberate methodological choice, developed jointly by all co-authors across their respective fields of expertise, under the technical supervision of an experienced university research librarian. We opted to prioritise sensitivity over precision, given the exploratory/qualitative nature of this review, and would therefore prefer to maintain the search strategy as currently reported.

- “For selection and charting, it would help to prespecify the level of agreement expected at the pilot stage before full screening proceeds, and to clarify how the charting form will accommodate conceptual and first person works, for which the field on sociodemographic characteristics of the sample does not apply. Given the interpretive nature of the subject, a brief statement on reviewer disciplinary background and positionality would add welcome transparency.”

We thank the reviewer for these valuable comments. We have added the level of agreement expected at the pilot stage for both the selection and charting phases before proceeding with the full screening. We have also clarified how the charting form will accommodate conceptual and first-person works. Finally, we have added a “Reviewers’ Positionality” paragraph to the Methods section.

- “Finally, a few reporting details. There is no protocol specific extension of PRISMA-ScR, so the mention of PRISMA-ScR P is better replaced by the JBI best practice guidance already cited as reference 39. As BMJ Open asks protocols to report study dates, please add the anticipated start and completion of screening, extraction and synthesis, together with the OSF registration date and a policy for documenting deviations. A completed PRISMA-ScR checklist would be welcome, and a language check is advisable, since slips such as expecially, disproportionaly and an experienced university research remain.”

We thank the reviewer for these helpful suggestions. Regarding the comments related to the PRISMA-ScR and the related checklist, please refer to our replies above addressing similar points. As stated above, we have added the planned timing for further review steps to the full text (Methods section, "Search strategy" paragraph), as well as the OSF registration date. We have also introduced a policy for documenting deviations, in a new paragraph in the Methods section entitled "Protocol amendments". We have also corrected some mistakes that we had unintentionally left unresolved.
