# Supplementary material for: Understanding epistemic injustice among individuals with psychotic experiences: a scoping review protocol
Source: BMJ Open. 2026 Oct 26;16(10):e125288. doi: 10.1136/bmjopen-2026-125288 (PMC13711525; doi:10.1136/bmjopen-2026-125288)
Supplement: Supplementary file 3 [file bmjopen-16-10-s001.docx]

**Preliminary search conducted on: 26 June 2026**

**Filter applied across all databases: publication date from 2007 onwards.**

**MEDLINE, Interface Ovid, search string**

(("epistemic injustice" or "testimonial injustice" or "hermeneutical injustice" or "epistemic oppression" or "epistemic marginalization" or "epistemic marginalisation" or "epistemic exclusion" or "epistemic violence" or "epistemic harm" or "epistemic silencing" or "epistemic invalidation" or "epistemic exploitation" or "silencing" or "credibility deficit" or "testimonial smothering" or "testimonial quieting" or "interpretive injustice" or "hermeneutical gap" or "lived experience" or "patient voice" or "experiential knowledge" or "dismissal" or objectification or "epistemology of psychiatry" or "medical gaslighting") OR (feminism/ or exp patient rights/ or exp social justice/ or exp antiracism/ or exp environmental justice/)) AND (("psychosis" or "psychotic" or "psychot*" or "schizophrenia" or "schizoaffective" or "first episode psychosis" or "early psychosis" or "delusion*" or "hallucinat*" or "voice hearing" or "hearing voices" or "auditory verbal hallucinations" or AVH or "unusual experiences" or "anomalous experiences" or "lived experience of psychosis" or "minimal self disturbance" or "paranoia" or "persecution") OR (exp "schizophrenia spectrum and other psychotic disorders"/ or exp affective disorders, psychotic/ or exp capgras syndrome/ or exp delusional parasitosis/ or exp morgellons disease/ or exp paranoid disorders/ or exp psychotic disorders/ or exp psychoses, substance-induced/ or exp psychoses, alcoholic/ or exp schizophrenia/ or exp schizophrenia, catatonic/ or exp schizophrenia, disorganized/ or exp schizophrenia, paranoid/ or exp schizophrenia, treatment-resistant/ or exp shared paranoid disorder/))  [mp=title, book title, abstract, original title, name of substance word, subject heading word, floating sub-heading word, keyword heading word, organism supplementary concept word, protocol supplementary concept word, rare disease supplementary concept word, unique identifier, synonyms, population supplementary concept word, anatomy supplementary concept word]

**PsychINFO, Interface Ovid, search string**

(("epistemic injustice" or "testimonial injustice" or "hermeneutical injustice" or "epistemic oppression" or "epistemic marginalization" or "epistemic marginalisation" or "epistemic exclusion" or "epistemic violence" or "epistemic harm" or "epistemic silencing" or "epistemic invalidation" or "epistemic exploitation" or "silencing" or "credibility deficit" or "testimonial smothering" or "testimonial quieting" or "interpretive injustice" or "hermeneutical gap" or "lived experience" or "patient voice" or "experiential knowledge" or "dismissal" or objectification or "epistemology of psychiatry" or "medical gaslighting") OR (exp hermeneutics/ or exp racism/ or exp religious prejudices/ or exp sexism/ or exp age discrimination/ or exp disability discrimination/ or exp ethnic bias/ or exp implicit bias/ or exp stereotyped attitudes/ or exp paternalism/ )) AND (("psychosis" or "psychotic" or "psychot*" or "schizophrenia" or "schizoaffective" or "first episode psychosis" or "early psychosis" or "delusion*" or "hallucinat*" or "voice hearing" or "hearing voices" or "auditory verbal hallucinations" or AVH or "unusual experiences" or "anomalous experiences" or "lived experience of psychosis" or "minimal self disturbance" or "paranoia" or "persecution") OR (exp Experimental Psychosis/ or exp Postpartum Psychosis/ or exp Childhood Onset Psychosis/ or exp Psychosis/ or exp Affective Psychosis/ or exp Chronic Psychosis/ or exp Reactive Psychosis/ or exp Paranoid Psychosis/)) [mp=title, abstract, heading word, table of contents, key concepts, original title, tests & measures, mesh word]

**CINAHL, Interface EBSCOhost, search string**

[(("psychosis" or "psychotic" or "psychot*" or "schizophrenia" or "schizoaffective" or "first episode psychosis" or "early psychosis" or "delusion*" or "hallucinat*" or "voice hearing" or "hearing voices" or "auditory verbal hallucinations" or AVH or "unusual experiences" or "anomalous experiences" or "lived experience of psychosis" or "minimal self disturbance" or "paranoia" or "persecution") OR (MM "Psychotic Disorders+" OR MM "Postpartum Psychosis" OR MM "ICU Psychosis" OR MM "Psychoses, Substance-Induced+" OR MM "Psychoses, Alcoholic+" OR MM "Organic Mental Disorders, Psychotic+" OR MM "Paranoid Disorders" OR MM "Affective Disorders, Psychotic+" OR MM "Schizoaffective Disorder" OR MH "Bipolar Disorder" OR MH "Dementia, Presenile" OR MH "Dementia, Senile"))](https://research.ebsco.com/c/bz7hud/search/advanced-results?db=rzh&expanders=concept&limiters=None&q=%28%28%22psychosis%22%20or%20%22psychotic%22%20or%20%22psychot%2A%22%20or%20%22schizophrenia%22%20or%20%22schizoaffective%22%20or%20%22first%20episode%20psychosis%22%20or%20%22early%20psychosis%22%20or%20%22delusion%2A%22%20or%20%22hallucinat%2A%22%20or%20%22voice%20hearing%22%20or%20%22hearing%20voices%22%20or%20%22auditory%20verbal%20hallucinations%22%20or%20AVH%20or%20%22unusual%20experiences%22%20or%20%22anomalous%20experiences%22%20or%20%22lived%20experience%20of%20psychosis%22%20or%20%22minimal%20self%20disturbance%22%20or%20%22paranoia%22%20or%20%22persecution%22%29%29%20OR%20%28MM%20%22Psychotic%20Disorders%2B%22%20OR%20MM%20%22Postpartum%20Psychosis%22%20OR%20MM%20%22Psychoses%2C%20Substance-Induced%22%20OR%20MM%20%22Psychoses%2C%20Alcoholic%22%20OR%20MM%20%22Paranoid%20Disorders%22%20OR%20MM%20%22Schizoaffective%20Disorder%22%29&searchMode=boolean&searchQueryId=sq%3A6c54a114-79fd-4032-998c-e522cc88b1d4&sort=relevance&userDirectAction=true) AND ("epistemic injustice" or "testimonial injustice" or "hermeneutical injustice" or "epistemic oppression" or "epistemic marginalization" or "epistemic marginalisation" or "epistemic exclusion" or "epistemic violence" or "epistemic harm" or "epistemic silencing" or "epistemic invalidation" or "epistemic exploitation" or "silencing" or "credibility deficit" or "testimonial smothering" or "testimonial quieting" or "interpretive injustice" or "hermeneutical gap" or "lived experience" or "patient voice" or "experiential knowledge" or "dismissal" or objectification or "epistemology of psychiatry" or "medical gaslighting")

**Web of Science, Core Collection, Interface Clarivate, default/basic search, search string**

(("epistemic injustice" or "testimonial injustice" or "hermeneutical injustice" or "epistemic oppression" or "epistemic marginalization" or "epistemic marginalisation" or "epistemic exclusion" or "epistemic violence" or "epistemic harm" or "epistemic silencing" or "epistemic invalidation" or "epistemic exploitation" or "silencing" or "credibility deficit" or "testimonial smothering" or "testimonial quieting" or "interpretive injustice" or "hermeneutical gap" or "lived experience" or "patient voice" or "experiential knowledge" or "dismissal" or objectification or "epistemology of psychiatry" or "medical gaslighting") AND ("psychosis" or "psychotic" or "psychot*" or "schizophrenia" or "schizoaffective" or "first episode psychosis" or "early psychosis" or "delusion*" or "hallucinat*" or "voice hearing" or "hearing voices" or "auditory verbal hallucinations" or AVH or "unusual experiences" or "anomalous experiences" or "lived experience of psychosis" or "minimal self disturbance" or "paranoia" or "persecution"))

**PhilPapers, simple search, string**

'epistemic injustice testimonial hermeneutical AND psychosis schizophrenia delusion hallucination voice hearing unusual experiences paranoia'

**CORE, simple Boolean search, string**

(epistemic injustice OR testimonial injustice OR hermeneutical injustice) AND (psychosis)
