# Supplementary material for: Understanding epistemic injustice among individuals with psychotic experiences: a scoping review protocol
Source: BMJ Open. 2026 Oct 26;16(10):e125288. doi: 10.1136/bmjopen-2026-125288 (PMC13711525; doi:10.1136/bmjopen-2026-125288)
Supplement: Supplementary file 4 [file bmjopen-16-10-s003.docx]

# PRISMA-P (Preferred Reporting Items for Systematic review and Meta-Analysis Protocols) 2015 Checklist

We applied the PRISMA-P checklist to our scoping review protocol because, to the best of our knowledge, no specific checklist for scoping review protocols is currently available. Therefore, please note that, when checklist items refer to systematic reviews, we have interpreted them as referring to scoping reviews instead.

Recommended items to address in a systematic review protocol*

| Section & Topic | Item No. | Checklist Item | Information reported | | Line number(s) |
| --- | --- | --- | --- | --- | --- |
| ADMINISTRATIVE INFO |  |  |  |  |  |
| Title |  |  |  |  |  |
| Identification | 1a | Identify the report as a protocol of a systematic review | Yes | | Title page (page 1) |
| Update | 1b | If the protocol is for an update of a previous systematic review, identify as such | No | | Not an update |
| Registration |  |  |  |  |  |
|  | 2 | If registered, provide the name of the registry (such as PROSPERO) and registration number | Yes | | Abstract (page 2); Methods (page 7) |
| Authors |  |  |  |  |  |
| Contact | 3a | Provide name, institutional affiliation, e-mail address of all protocol authors; provide physical mailing address of corresponding author | Yes | | Title page (page 1) |
| Contributions | 3b | Describe contributions of protocol authors and identify the guarantor of the review | Yes | | Ethics and dissemination (pages 10-11) |
| Amendments |  |  |  |  |  |
| Amendments | 4 | If the protocol represents an amendment of a previously completed or published protocol, identify as such and list changes; otherwise, state plan for documenting important protocol amendments | No | | Not an amendment |
| Support |  |  |  |  |  |
| Sources | 5a | Indicate sources of financial or other support for the review | Yes | | Support/funding paragraph (page 10) |
| Sponsor | 5b | Provide name for the review funder and/or sponsor | Yes | | Support/funding paragraph (page 10) |
| Role of Sponsor or Funder | 5c | Describe roles of funder(s), sponsor(s), and/or institution(s), if any, in developing the protocol | Yes | | Support/funding paragraph (page 10) |
| INTRODUCTION |  |  |  |  |  |
| Rationale |  |  |  |  |  |
| Rationale | 6 | Describe the rationale for the review in the context of what is already known | Yes | | Introduction (pages 2-5) |
| Objectives |  |  |  |  |  |
| Objectives | 7 | Provide an explicit statement of the question(s) the review will address with reference to participants, interventions, comparators, and outcomes (PICO) | Yes | | Objectives paragraph (page 5) |
| METHODS |  |  |  |  |  |
| Eligibility Criteria |  |  |  |  |  |
| Eligibility criteria | 8 | Specify the study characteristics (such as PICO, study design, setting, time frame) and report characteristics (such as years considered, language, publication status) to be used as criteria for eligibility for the review | Yes | | Eligibility criteria paragraph (pages 5-6) |
| Information Sources |  |  |  |  |  |
| Information sources | 9 | Describe all intended information sources (such as electronic databases, contact with study authors, trial registers or other grey literature sources) with planned dates of coverage | Yes | | Search strategy paragraph (pages 7-8) |
| Search Strategy |  |  |  |  |  |
| Search strategy | 10 | Present draft of search strategy to be used for at least one electronic database, including planned limits, such that it could be repeated | Yes | | Search strategy paragraph (pages 7-8); Appendix I |
| Study Records |  |  |  |  |  |
| Data management | 11a | Describe the mechanism(s) that will be used to manage records and data throughout the review | Yes | | Study/source of evidence selections paragraph (page 8) |
| Selection process | 11b | State the process that will be used for selecting studies (such as two independent reviewers) through each phase of the review (that is, screening, eligibility and inclusion in meta-analysis) | Yes | | Study/source of evidence selections paragraph (page 8) |
| Data collection process | 11c | Describe planned method of extracting data from reports (such as piloting forms, done independently, in duplicate), any processes for obtaining and confirming data from investigators | Yes | | Data Extraction and Charting paragraph (pages 8-9) |
| Data Items |  |  |  |  |  |
| Data items | 12 | List and define all variables for which data will be sought (such as PICO items, funding sources), any pre-planned data assumptions and simplifications | Yes | | Data Extraction and Charting paragraph (page 8) |
| Outcomes and Prioritization |  |  |  |  |  |
| Outcomes and prioritization | 13 | List and define all outcomes for which data will be sought, including prioritization of main and additional outcomes, with rationale | Yes | | Review questions paragraph (pages 5-6) |
| Risk of Bias |  |  |  |  |  |
| Risk of bias in individual studies | 14 | Describe anticipated methods for assessing risk of bias of individual studies, including whether this will be done at the outcome or study level, or both; state how this information will be used in data synthesis | No | | Not applicable, as this was not required for a scoping review. |
| Data Synthesis |  |  |  |  |  |
| Data synthesis | 15a | Describe criteria under which study data will be quantitatively synthesised | No | | Not applicable, as this was not required for a scoping review. |
|  | 15b | If data are appropriate for quantitative synthesis, describe planned summary measures, methods of handling data and methods of combining data from studies, including any planned exploration of consistency (such as I^2^, Kendall’s τ) | No | | Not applicable, as this was not required for a scoping review. |
|  | 15c | Describe any proposed additional analyses (such as sensitivity or subgroup analyses, meta-regression) | No | | Not applicable, as this was not required for a scoping review. |
|  | 15d | If quantitative synthesis is not appropriate, describe the type of summary planned | Yes | | Data analysis and presentation paragraph (page 9) |
| Meta-bias(es) |  |  |  |  |  |
| Meta-bias(es) | 16 | Specify any planned assessment of meta-bias(es) (such as publication bias across studies, selective reporting within studies) | No | | Not applicable, as this was not required for a scoping review. |
| Confidence in Evidence |  |  |  |  |  |
| Confidence in cumulative evidence | 17 | Describe how the strength of the body of evidence will be assessed (such as GRADE) | No | | Not applicable, as this was not required for a scoping review. |

*It is strongly recommended that this checklist be read in conjunction with the PRISMA-P Explanation and Elaboration for important clarification on the items. Amendments to a review protocol should be tracked and dated. The copyright for PRISMA-P (including checklist) is held by the PRISMA-P Group and is distributed under a Creative Commons Attribution Licence 4.0.

From: *Shamseer L, Moher D, Clarke M, Ghersi D, Liberati A, Petticrew M, Shekelle P, Stewart L, PRISMA-P Group. Preferred reporting items for systematic review and meta-analysis protocols (PRISMA-P) 2015: elaboration and explanation. BMJ. 2015 Jan 2;349(jan02 1):g7647.*
